# Tumor mutational burden status and clinical characteristics of invasive lobular carcinoma of the breast

**DOI:** 10.1007/s12282-025-01706-6

**Published:** 2025-05-02

**Authors:** Yuko Takano, Kazuyuki Mizuno, Madoka Iwase, Sachi Morita, Nao Torii, Toyone Kikumori, Yuichi Ando

**Affiliations:** 1 Department of Clinical Oncology and Chemotherapy, Nagoya University Hospital, 65 Tsurumai-Cho, Showa-Ku, Nagoya, Aichi 466-8550 Japan; 2 Department of Breast and Endocrine Surgery, Nagoya University Hospital, 65 Tsurumai-Cho, Showa-Ku, Nagoya, Aichi 466-8550 Japan

**Keywords:** High tumor mutational burden, Invasive lobular carcinoma, Invasive ductal carcinoma

## Abstract

**Background:**

High tumor mutational burden (TMB-H) is an established biomarker for a favorable response to immune checkpoint inhibitors. However, tumor mutational burden (TMB) in invasive ductal carcinoma (IDC) and invasive lobular carcinoma (ILC) has not been sufficiently investigated.

**Methods:**

We collected data of patients with ILC or IDC from the Center for Cancer Genomics and Advanced Therapeutics database between June 2019 and August 2023. Furthermore, we examined the clinicopathological factors and TMB status.

**Results:**

Patients with ILC (n = 170) had a median TMB score of 4.00 mut/Mb (interquartile range, 2.00–7.14 mut/Mb), whereas those with IDC (n = 2598) had a score of 3.90 mut/Mb (2.00–6.00 mut/Mb). TMB-H was more common in patients with ILC than in those with IDC (18.2% vs. 10.1%, *P* < 0.001), particularly in the ER+ /HER2− subtype. Multivariate analysis revealed that the pathological diagnosis of ILC (*P* = 0.006), tissue samples collected from metastatic sites (*P* < 0.001), and older age (50 years, *P* < 0.001) were independent factors for TMB-H.

**Conclusions:**

Patients with ILC were more likely to have TMB-H than those with IDC. The findings of this study would be invaluable in selecting treatment strategies for patients with ILC.

## Introduction

Invasive lobular carcinoma (ILC) is the second most common histological type of invasive breast cancer next to invasive ductal carcinoma (IDC); it accounts for 5–15% of all breast cancer cases [1, 2]. ILC and IDC differ in terms of clinicopathological factors, features, and prognosis [1, 2]. Patients with ILC and those with IDC are usually treated similarly, depending on the molecular classification: anthracycline- and taxane-based chemotherapy, hormone therapy for estrogen receptor (ER)- and/or progesterone receptor (PgR) receptor-positive subtypes, and anti-HER2 therapy for HER2 + subtypes. Poly-adenosine diphosphate ribose polymerase inhibitors have been approved for the treatment of patients with pathogenic germline variants in *BRCA1/2* genes with the HER2− subtype. In addition, immune checkpoint inhibitors (ICIs) have recently been introduced into the standard of care for antiprogramed cell death ligand 1-positive recurrent triple-negative breast cancer (TNBC) [3]. They have also been approved for the treatment of solid tumors with a tumor mutational burden (TMB) status of ≥ 10 mutations/megabases (mut/Mb, high TMB [TMB-H]), the latter being a tumor agnostic indicator [4].

A high TMB status is recognized as a positive biomarker for better clinical response to ICIs in the treatment of many cancers, including breast cancer [4]. With the widespread use of comprehensive genomic profiling (CGP) testing in clinical practice, the TMB status as a biomarker of the therapeutic efficacy of ICIs had gained considerable interest among clinicians. The percentage of patients with TMB-H has been reported to be 3–10% in all breast cancer cases [5]; however, the distribution of TMB and the percentage of patients with TMB-H has not been sufficiently investigated across the molecular and histological subtypes. Therefore, this study investigated the TMB status and clinical characteristics of patients with various molecular and histological subtypes of breast cancer, particularly ILC, using a large nationwide database of genomic information in Japan.

## Patients and methods

To explore the TMB status and clinical characteristics of patients with various molecular and histological subtypes of breast cancer, we evaluated the genomic profiles of patients with breast cancer who had been registered in the Center for Cancer Genomics and Advanced Therapeutics (C-CAT) database between June 2019 and August 2023. The C-CAT database is a large-scale nationwide database that aggregates the data of the CGP test (panel), which is covered by public health insurance in Japan. Nearly all the patients in the study were highly likely to be Japanese.

The database was searched on March 12, 2024. Patients with IDC or ILC were extracted based on the detailed pathological diagnosis registered in the database. Only patients who received two tissue-based panels, the OncoGuide^™^ NCC Oncopanel System (NOP) and FoundationOne^®^ CDx Cancer Genomic Profile (F1 CDx), were included in the analysis. Those tested using other panels (FoundationOne^®^ CDx Liquid, Guardant 360^®^CDx, GenMineTOP^®^) were excluded. The following data were obtained from the database: age, gender, histological subtype, test panel, date and sample collection site, treatments before and after sample collection, ER and PgR status, HER2 status, pathogenic variants in the germline *BRCA1/2* genes, TMB score, and microsatellite instability status. ER and PgR were registered in the database only as positive or negative, unknown or untested. HER2 was defined as HER2 positive when Immunohistochemical staining (IHC) was 3 + or positive by HER2 in-situ Hybridization (ISH) and HER2 negative when HER2 IHC was 0, 1 +, or 2 + and negative by ISH. TMB was defined as the total number of synonymous or nonsynonymous somatic mutations in the target regions of the tumor genome and expressed as mutations per 1 megabase (mut/Mb). Owing to the log-normal distribution of the TMB score, the TMB score variable was log-transformed. A TMB status of 10 mut/Mb or greater was defined as TMB-H.

The differences in the patient clinicopathological factors between IDC and ILC were compared using the Mann–Whitney *U* test or the chi-squared test. The influences of cancer subtype, test panel, and sample collection site on the TMB score or the percentage of patients with TMB-H were compared using the Mann–Whitney *U* test. All statistical analyses were conducted using IBM SPSS Statistics version 29.0 (IBM Japan Ltd., Tokyo, Japan) and R version 4.3.1 [6].* P*-values < 0.05 were considered statistically significant.

This study was conducted in accordance with the Ethical Guidelines for Medical and Biological Research Involving Human Subjects (Ministry of Health, Labour and Welfare, Japan) and the Declaration of Helsinki. Furthermore, it was approved by the Institutional Review Boards of Nagoya University Hospital (approval no.: 2022–0025) and C-CAT (C-CAT control number: CDU2022-030 N). All participants provided written consent for the use of their genomic data before enrollment into the C-CAT database.

## Results

**Fig. 1:**
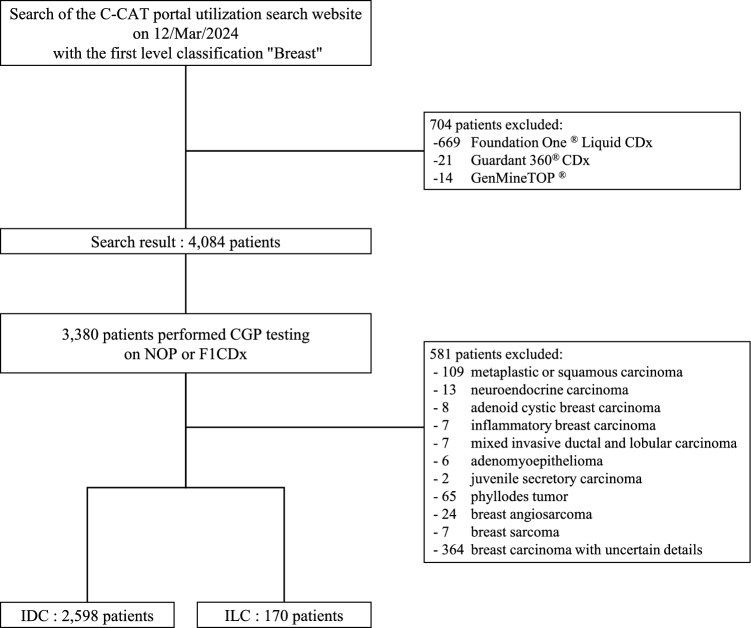
Composition of patients of study criteria

Of the 4084 patients with breast cancer registered in the C-CAT database, 3380 patients had been tested for NOP or F1 CDx. We extracted 2598 and 170 patients with IDC and ILC, respectively, for analysis (Fig. 1, Table 1). Compared with the IDC group, the ILC group had a higher proportion of patients who were older and had the luminal type (ER+/HER2−) (Table 1).

**Table 1:** Patient characteristics

	IDC (n = 2598)		ILC (n = 170)		*P* value
Age-median (range)	54	(26–91)	60	(27–82)	< 0.001
Sex-n (%)					
Female	2582	(99.4)	168	(98.8)	0.698
Male	16	(0.6)	2	(1.2)	
Subtype-n (%)					< 0.001
Luminal (ER+, HER2−)	1264	(48.7)	118	(69.4)	
ER+, PgR+, HER2−	878	(33.8)	68	(40.0)	
ER+, PgR−, HER2−	376	(14.5)	49	(28.8)	
ER+, PgR unknown, HER2−	10	(0.4)	1	(0.6)	
Luminal-HER2 (ER+, HER2+)	193	(7.4)	4	(2.4)	
HER2 (ER−, HER2+)	142	(5.5)	2	(1.2)	
Triple negative (ER−, HER2−)	856	(32.9)	39	(23.5)	
Unknown	143	(5.5)	7	(4.1)	
Panel-n (%)					0.255
OncoGuide^™^ NCC oncopanel system	321	(12.4)	16	(9.4)	
FoundationOne^® ^CDx	2277	(87.6)	154	(90.6)	
Sample collection site-n (%)					0.812
Primary breast	1,377	(53.0)	87	(51.2)	
Metastatic site	1,219	(46.9)	81	(47.6)	
Unknown	2	(0.1)	2	(1.2)	
Germline *BRCA1* PV-n (%)					0.324
Positive	73*	(2.8)	1	(0.6)	
Negative	1546	(59.5)	108	(63.5)	
VUS	13	(0.5)	0	(0.0)	
Unknown	966	(37.2)	61	(35.9)	
Germline *BRCA2* PV-n (%)					0.206
Positive	115*	(4.4)	2	(1.2)	
Negative	1502	(57.8)	105	(61.8)	
VUS	16	(0.6)	0	(0.0)	
Unknown	965	(37.1)	63	(37.1)	
MSI-status-n (%)					0.859
High	10	(0.4)	1	(0.6)	
Non-high or stable	2288	(88.1)	151	(88.8)	
Unknown or cannot be determined	300	(11.5)	18	(10.6)	

^*^Two patients had both of *BRCA1* and *BRCA2* pathogenic variants

*IDC* Invasive ductal carcinoma, *ILC* invasive lobular carcinoma, *ER* estrogen receptor, *PgR* progesterone receptor, *HER2* human epidermal growth factor receptor type 2, *PV* pathogenic variants, *VUS* variant with unknown significance, *MSI* microsatellite instability

**Fig. 2:**
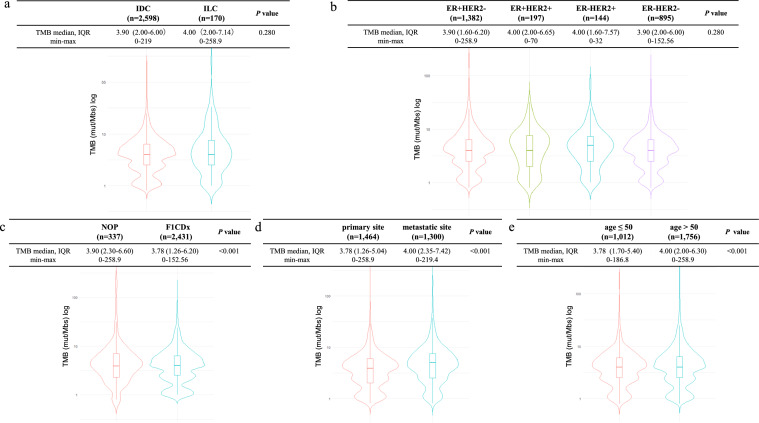
Distribution and median of TMB in each subgroup. The y-axis is displayed in logarithmic form. The difference between IDCs and ILCs (**a**), between subtypes (**b**), between test panels (**c**), sample collection sites (**d**), or between ages (< 50 vs. > 50 years) (**e**) are shown

The median TMB scores for the IDC and ILC groups were 3.90 and 4.00 mut/Mb, respectively (Fig. 2a, Table 2). When tested with NOP, the ILC group (8.95 mut/Mb) exhibited a higher score than the IDC group (Table 2). There were no significant differences in the distribution of TMB by subtype, test panel (NOP vs. F1 CDx), or specimen collection site (primary or metastatic site; Fig. 2b–d). Compared with age, the distribution of TMB was significantly higher for patients aged > 50 years than for those aged < 50 years. (median TMB score 3.78 vs. 4.00; p < 0.001; Fig. 2e). In addition, while comparing IDC and ILC for each patient characteristics, TMB was higher in IDC for positive g*BRCA*2 pathogenic variants (median TMB 6.00 vs. 2.26 mut/Mb, p = 0.042) and in ILC for negative pathogenic variants of g*BRCA*2 (median TMB 3.78 vs 4.00 mut/Mb, p = 0.017; Table 2).

**Table 2:** Distribution of TMB between IDC and ILC

	IDC (n = 2598)			ILC (n = 170)			*P* value
	n	TMB median, (IQR)		n	TMB median, (IQR)		
All	2598	3.90	(2.00–6.00)	170	4.00	(2.00–7.14)	0.280
Age (year)							
≤ 50	983	3.78	(2.00–5.40)	29	2.52	(1.26–6.5)	0.639
> 50	1615	4.00	(2.00–6.30)	141	4.00	(2.00–7.57)	0.284
Subtypes							
ER+ HER2−	1,264	3.84	(1.60–6.00)	118	4.00	(2.00–7.57)	0.158
ER+PgR+HER2−	878	3.78	(1.26–6.00)	68	3.90	(1.26–6.30)	0.676
ER+PgR−HER2−	376	4.00	(2.30–6.30)	49	4.00	(2.52–10.50)	0.251
ER−HER2+	142	4.00	(1.51–7.57)	2	3.00	(2.00–NA)	0.598
ER+HER2+	193	4.00	(2.00–6.30)	4	5.50	(1.75–13.75)	0.607
ER−HER2−	856	3.90	(2.00–6.00)	39	4.00	(2.00–7.57)	0.350
Sample collection site							
Primary site	1337	3.78	(1.26–5.04)	87	3.78	(1.26–6.00)	0.681
Metastatic site	1219	4.00	(2.52–7.00)	81	4.00	(2.15–10.95)	0.316
Panels							
NOP	321	3.90	(2.30–6.20)	16	8.95	(4.68–25.18)	< 0.001
F1 CDx	2277	3.78	(2.00–6.00)	154	3.78	(1.26–6.30)	0.948
g*BRCA1* PV							
Positive	73	4.00	(2.00–6.30)	1	1.00	NA	0.216
Negative	1546	3.90	(2.00–6.00)	108	4.00	(2.00–8.00)	0.060
VUS	13	5.04	(2.75–6.3)	0	NA		NA
Unknown	899	3.90	(1.60–6.30)	50	3.78	(2.00–6.30)	0.996
g*BRCA2* PV							
Positive	115	6.00	(3.78–9.00)	2	2.26	(2.00-NA)	0.042
Negative	1502	3.78	(2.00–6.00)	105	4.00	(2.00–8.42)	0.017
VUS	16	5.00	(1.78–6.83)	0	NA		NA
Unknown	898	3.78	(1.60–6.20)	63	3.44	(2.00–6.23)	0.809

*TMB* tumor mutational burden, *IDC* Invasive ductal carcinoma, *ILC* Invasive lobular carcinoma, *ER* Estrogen receptor, *PgR* Progesterone receptor, *HER2* Human epidermal growth factor receptor type 2, *F1 CDx* Foundation One^®^ CDx, *NOP* OncoguideTM NCC Oncopanel system, g*BRCA1* germline *BRCA1*, g*BRCA2* germline *BRCA2*, *PV* pathogenic variants, *VUS* variant of uncertain significance, *NA* not applicable

**Fig. 3:**
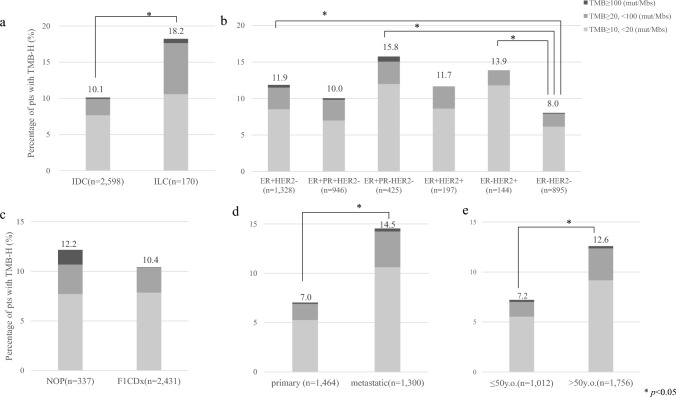
Percentage of patients with TMB-H. The difference between IDCs and ILCs (**a**), between subtypes (**b**), between test panels (**c**), sample collection sites (**d**), or between ages (≤ 50 vs. > 50 years) (**e**) are shown

The numbers of TMB-H cases in the IDC and ILC groups were 263 (10.1%) and 31 (18.2%), respectively (Table 3, Fig. 3a), with the ILC group showing a significantly higher proportion. In particular, while comparing the numbers of ≥ 20 mut/Mb, 13 (7.6%) in ILC, and 64 (2.5%) in IDC, the proportion is particularly high in ILC (Fig. 3a). While comparing the numbers of TMB-H cases, 164 (11.9%) in ER+HER2−, 67 (15.8%) in the ER+PgR−HER2−, and 20 (13.9%) in the ER− HER2+ were significantly higher than the 72 (8.0%) of TNBC (Fig. 3b). There was no significant difference between the NOP and F1 CDx test panels; however, 103 (7.0%) and 189 (14.5%) specimens from the primary site and metastatic sites were TMB-H, and 73 (7.2%) and 221 (12.6%) of those aged ≤ 50 and > 50 were TMB-H. The proportion of TMB-H was significantly higher in specimens taken from metastatic sites and in those aged ≥ 50 (Fig. 3c, d).When the IDC and ILC groups were compared by subtype, 140 (11.2%) and 24 (20.3%) patients had ER+/HER2− breast cancer, respectively, with the latter group showing a significantly higher number of cases; however, for the other subtypes, no statistically significant difference was observed (Table 3). When comparison was performed based on sample collection site between the IDC and ILC groups, there were 95 (6.9%) and 8 (9.2%) patients, respectively, in whom samples were collected from the primary breast, and 168 (13.8%) and 21 (25.9%) patients, respectively, showed a significantly higher percentage of TMB-H in the ILC samples collected from metastatic sites for biopsy than in the IDC samples. When comparing IDC and ILC by test panels, 33 (10.3%) and 8 (50.0%) patients received NOP, whereas 230 (10.1%) and 23 (14.9%) received F1 CDx, respectively. When comparing IDC and ILC by age, 70 (7.1%) and 3 (10.3%) patients were aged below 50 years, whereas 193 (12.0%) and 28 (19.9%) patients were aged over 50 years, respectively. As a result, the proportion of patients with TMB-H who were tested using the NOP panel and who were aged over 50 years was significantly higher in the ILC than in the IDC group (Table 3). When comparing IDC and ILC by the g*BRCA1/2* status, 143 (9.2%) patients and 23 (21.3%), respectively, had negative for g*BRCA1* pathogenic variants and 134 (8.9%) and 23 (21.9%) patients, respectively, had negative for g*BRCA2* pathogenic variants, with the ILC group showing a significantly higher proportion of TMB-H cases (i.e., TMB-H cases within different cancer groups, such as ILC and IDC).

**Table 3:** Percentage of patients with TMB-H

	IDC + ILC		IDC		ILC		*P* value
	Total	TMB-H n (%)	Total	TMB-H n (%)	Total	TMB-H n (%)	
All	2,768	294 (10.6)	2,598	263 (10.1)	170	31 (18.2)	< 0.001
Subtype							
ER+, HER2−	1,382	164 (11.9)	1,264	140 (11.1)	118	24 (20.3)	0.003
ER+, PgR+, HER2−	946	95 (10.0)	878	85 (9.7)	68	10 (14.7)	0.184
ER+, PgR−, HER2−	425	67 (15.7)	376	53 (14.1)	49	14 (28.6)	0.009
ER+, HER2+	197	23 (11.7)	193	22 (11.4)	4	1 (25.0)	0.402
ER−, HER2+	144	20 (13.9)	142	20 (14.1)	2	0 (0)	1.000
ER−, HER2−	895	72 (8.0)	856	66 (7.7)	39	6 (15.4)	0.085
Sample collection site							
Primary site	1,424	103 (7.0)	1,377	95 (6.9)	87	8 (9.2)	0.435
Metastatic site	1,300	189 (14.5)	1,219	168 (13.8)	81	21 (25.9)	0.008
Panel							
NOP	337	41 (12.2)	321	33 (10.3)	16	8 (50.0)	< 0.001
F1 CDx	2,431	253 (10.4)	2,277	230 (10.1)	154	23 (14.9)	0.057
Age							
≤ 50	1,012	73 (7.2)	983	70 (7.1)	29	3 (10.3)	0.508
> 50	1,756	221 (12.6)	1615	193 (12.0)	141	28 (19.9)	0.007
g*BRCA1* PV							
Positive	74	5 (6.8)	73	5 (6.8)	1	0(0)	0.786
Negative	1,654	166(10.0)	1,546	143 (9.2)	108	23 (21.3)	< 0.001
VUS	13	1(7.7)	13	1 (7.7)	0	0 (NA)	NA
Unknown	949	112 (11.7)	966	104(11.6)	63	8(13.3)	0.683
g*BRCA2* PV							
Positive	117	15 (12.8)	115	15 (13.0)	2	0(0)	0.584
Negative	1,607	157 (9.8)	1,502	134 (8.9)	105	23 (21.9)	< 0.001
VUS	16	2 (12.5)	16	2 (12.5)	0	0 (NA)	NA
Unknown	961	114 (11.9)	965	106 (11.8)	63	8 (13.8)	0.648

*IDC* Invasive ductal carcinoma, *ILC* invasive lobular carcinoma, *ER* estrogen receptor, *PgR* progesterone receptor, *HER2* human epidermal growth factor receptor type 2, *NOP* OncoGuideTM NCC Oncopanel System, *F1 CDx* FoundationOne^®^ CDx, *PV* pathogenic variants, *VUS* variant of uncertain significance, NA not applicable

To evaluate the impact of previous treatment on the proportion of TMB-H cases, we compared the samples collected before and after drug therapy in terms of the proportion of TMB-H cases. We found that 20 (13.2%) and 274 (10.5%) cases in the samples collected before and after therapy (*P* = 0.282), respectively, had TMB-H, indicating that the treatment exerted no effect. According to treatment, TMB-H was observed in 104 (8.9%) and 141 (11.4%) cases in the samples collected before and after endocrine therapy (*P* = 0.046), 24 (11.1%) and 221 (10.1%) in the samples collected before and after chemotherapy (*P* = 0.639), and 222 (10.1%) and 221 (10.1%) in the samples collected before and after anti-HER2 therapy (*P* = 0.639), respectively. As regards the ER+/HER2− subtype, there were 30 (13.8%) and 105 (10.5%) cases before and after endocrine therapy, respectively (*P* = 0.180), whereas for the ER−/HER2− subtype, there were 47 (6.7%) and 18 (18.9%) cases, respectively (*P* < 0.001).

Table 4 presents the adjusted odds ratios and 95% confidence intervals (CI) from the logistic regression analysis adjusted for the aforementioned factors in patients with IDC and ILC (Table 4). The adjusted odds ratios (95% CI) for age ≥ 50 years, ILC, and sample collection from metastatic sites were 1.691 (1.264–2.263), 1.813 (1.181–2.783), and 2.109 (1.623–2.741), respectively. Meanwhile, subtype, test panel, and g*BRCA1/2* were not related to the proportion of TMB-H cases.

**Table 4:** Multivariate analysis of percentage of patients with TMB-H

Variable	Category	Adjusted OR	95% CI	*P* value
Age	≤ 50 (reference)			
	> 50	1.691	1.264–2.263	< 0.001
Type	IDC (reference)			
	ILC	1.813	1.181–2.783	0.006
Subtype	ER+, HER2− (reference)			0.211
	ER−, HER2+	1.289	0.759–2.189	0.348
	ER+, HER2+	0.973	0.595–1.591	0.913
	ER−, HER2−	0.747	0.553–1.011	0.059
	Unknown	0.661	0.290–1.509	0.326
Sample collection site	Primary site (reference)			
	Metastatic site	2.109	1.623–2.741	< 0.001
Panel	F1 CDx (reference)			
	NOP	1.140	0.791–1.641	0.482
g*BRCA1* PV	Negative (reference)			
	Positive	0.949	0.369–2.436	0.913
	VUS	0.625	0.061–6.411	0.692
	Unknown	3.587	0.708–18.175	0.123
g*BRCA2* PV	Negative (reference)			
	Positive	1.166	0.633–2.150	0.622
	VUS	2.291	0.416–12.616	0.341
	Unknown	1.027	1.015–1.039	0.125

*TMB-H* tumor mutational burden high, *IDC* Invasive ductal carcinoma, *ILC* Invasive lobular carcinoma, *ER* Estrogen receptor, *HER2* human epidermal growth factor type2, *NOP* OncoGuideTM NCC Oncopanel System, *F1 CDx* FoundationOne^®^ CDx, g*BRCA1* germline *BRCA1*, g*BRCA2* germline *BRCA2*, *PV* pathogenic variants, *VUS* variant of uncertain significance

## Discussion

In this study, the median TMB scores were 4.00 and 3.90 (mut/Mb) for the ILC and IDC groups, respectively, and these values did not differ between ILCs and IDCs when evaluated by the subtype, test panel, age, or sample collection site. Furthermore, the proportion of TMB-H cases was statistically significantly higher in the ILC than in the IDC group (18.2% vs. 10.1%, respectively). Moreover, the proportion of TMB-H cases in the ILC group was particularly high among those with the ER+/HER2− subtype and in whom metastatic lesion was the sample collection site. In addition, the proportion of TMB-H cases was higher among those with the ER +/HER2− and ER−/HER2 + subtypes than in the TNBC and to be higher in cases sampled from metastatic sites and in those aged 50 years or older. On the other hand, when comparing ILC and IDC, there was no difference in the distribution of TMB scores between ILC and IDC, expect for *BRCA1/2* pathogenic variant -negative cases or those tested by NOP. In other words, except for *BRCA 1/2* pathogenic variant-negative cases, there is no difference in the distribution of TMB between ILC and IDC, but there is a special population of TMB-H cases that is found more frequently in ILC than in IDC. This cannot be predicted by clinical factors alone, and it is necessary to predict based on factors such as gene alterations.

A high TMB is associated with a high neoantigen load, making the tumor in high immunogenic conditions. Compared with immunogenic tumors, such as skin squamous cell carcinoma (45.2 mut/Mb), melanoma (14.4 mut/Mb), and non-small cell lung carcinoma (8.1 mut/Mb), the TMB scores in breast cancer were reportedly lower (3.6–3.8 mut/Mb) [5, 7]. TNBC has been reported to have a higher TMB score than ER+ or HER2+ cancers because of its high response to immunotherapy [8, 9]. Reportedly, TMB score is also high in ER+ HER2− breast cancer [10]. Herein, the proportion of TMB-H cases was higher in those with the ER+/HER2− subtype than in the TNBC cohort. This is thought to be due to genomic diversity in HR+HER2− breast cancer. Similarly, it was higher in the ILC than in the IDC group, which is consistent with the result of a previous study that included breast cancer cohorts [9]. The proportion of TMB-H was not affected by the treatment, but the proportion of TMB-H cases was found to be high in the TNBC cohort after hormone therapy. The reason why the TNBC cohort was administered hormone therapy is unknown, but this may indicate that the result of hormone therapy for HR+/HER2− breast cancer patients changing to TNBC and may be related to the intratumonal heterogeneity of breast cancer for TMB status.

More *PIK3 CA* mutations were observed in ILC than in IDC, and it has been reported that specific *PIK3 CA* mutations in ILC and metastatic lesions [1] induced mutations in APOBEC genes and that the presence of APOBEC gene mutations is related to TMB-H [11]. In this study, we examined the relationship between information obtained from clinical practice and TMB, and since we did not obtain information on gene alterations, the association between gene alterations and TMB scores was not evaluated; however, the differences in the molecular characteristics between IDC and ILC led to the difference in the proportion of TMB-H.

Meanwhile, when comparing by the sample collection site, the proportion of TMB-H was higher in the brain metastasis of lung cancer than in other metastatic sites [12, 13]. Although differences in the proportion of TMB-H may vary depending on the site from which the specimen was taken and on the type of cancer, the TMB is often higher at metastatic sites than at the primary site, even within breast cancer [9, 13, 14]. In this study, although the number of cases of brain metastasis was not enough to make comparisons, the proportion of TMB-H in metastatic lesions was higher than in primary tumors, indicating that there may be differences depending on the sample collection site. In patients without the pathogenic variant of g*BRCA1/2*, the TMB was higher in ILC than in IDC, whereas in those with the pathogenic variant, no difference was observed. However, in the multivariate analysis, the status of g*BRCA1/2* did not affect the proportion of TMB-H. Breast cancer with the *BRCA1/2* gene mutations is thought to have relatively high TMB [15]; however, the number of ILC cases with g*BRCA1/2* pathogenic variants is not enough to allow for sufficient consideration. ILC has more germline *CDH1* variants than IDC, still only around 0.54% [1]. Patients without pathogenic variants of g*BRCA1/2* should also include those with other germline gene variants associated with hereditary breast cancer; however, further investigation on the association between such germline variants and TMB-H is warranted.

The KEYNOTE-158 study confirmed the efficacy of pembrolizumab in the treatment of solid tumors with TMB-H, with an overall response rate of 29% [4]; however, it did not include patients with breast cancer. The Checkmate 848 trial was a phase II study that randomly assigned patients with tumor TMB-H and/or blood TMB-H solid tumors to the nivolumab (NIVO) + ipilimumab (IPI) therapy or NIVO monotherapy. The objective response rates for t-TMB-H were 38.6% (28.4–49.6) in the NIVO + IPI group and 29.8% (17.3–44.9) in the NIVO group. Of the 211 randomized patients in this study, 15 (7.1%) had breast cancer [16, 17]. The results of the TAPUR study confirmed the efficacy of pembrolizumab in the treatment of breast cancer with TMB-H (TNBC, 46%; HR+/HER2−, 43%), with disease control and response rates of 37% and 21%, respectively [18]. These suggest that patients with breast cancer with TMB-H may also benefit from ICI, even in other than TNBC, especially in ILC patients. Although some ILCs were highly immunogenic, this high immunogenicity does not necessarily correspond to TMB-H [19]. The efficacy of ICI in patients with TMB-H may be limited in ILCs that are not immunologically “hot,” and further investigation is needed.

This study has several limitations. First, compared with IDC, the number of ILC cases was extremely small, particularly ILC cases tested using the NOP or with g*BRCA1/2* pathogenic variants. Furthermore, the background of the patients who were tested may have greatly differed depending on the subtype as the tests were conducted under Japanese insurance reimbursement. For example, the proportion of TNBC patients was higher, and the proportion of HER2 + type patients was lower than the general population. Second, patients who had completed or were expected to complete the standard treatment were considered eligible for the tests. The small proportion of HER2 + types compared with the real world could be attributed to the fact that few clinicians were expected to benefit from the panel testing due to the already existing oncogene. Third, in this study, we excluded blood TMB to first elucidate tumor TMB. However, to the best of our knowledge, this is the first study to investigate in detail TMB in breast cancer patients using a public database in Japan.

In conclusion, this study demonstrated that the patients with ILC were more likely to have TMB-H than those with IDC. From the perspective of ICI therapy based on the TMB status, the findings would be invaluable in selecting treatment strategies for patients with ILC.

## Data Availability

The datasets generated and analyzed during the current study are available from the corresponding author on reasonable request.
